# Relationship between Highly Active Antiretroviral Therapy (HAART) and human papillomavirus type 16 (HPV 16) infection among women in Sub-Saharan Africa and public health implications: A systematic review

**DOI:** 10.1371/journal.pone.0213086

**Published:** 2019-03-11

**Authors:** Sonia Menon, Rodolfo Rossi, Mbabazi Kariisa, Sushama D. Acharya, Natasha Zdraveska, Sultan Mahmood, Steven Callens, Zacharie Ndizeye

**Affiliations:** 1 International Centre for Reproductive Health (ICRH), Ghent University, De Pintelaan, Ghent, Belgium; 2 Primary Health Care Services, International Committee of the Red Cross, Geneva, Switzerland; 3 March of Dimes Foundation, White Plains, New York, United States of America; 4 CDC Foundation, Atlanta, Georgia, United States of America; 5 Faculty of Pharmacy, Department of Clinical Pharmacy, Saints Cyril and Methodius University (Alumni), Skopje, Republic of Macedonia; 6 Massey University Alumni, Antwerp, Belgium; 7 Department of Internal Medicine & Infectious Diseases, University Hospital, Ghent, Belgium; 8 Community Medicine Department, Faculty of Medicine, University of Burundi, Bujumbura, Burundi; 9 Global Health Institute, Faculty of Medicine and Health Sciences, University of Antwerp, Antwerp, Belgium; Universita degli Studi di Roma Tor Vergata, ITALY

## Abstract

Invasive cervical cancer is the most prevalent cancer among women in Sub-Saharan Africa. In 2013, the World Health Organization (WHO) emitted recommendations to start Highly Active Antiretroviral Therapy (HAART) regardless of CD4 count. Although HAART has been shown to reduce the prevalence of high-risk human papillomavirus (HR-HPV) genotypes, it is unclear whether it confers a protective effect specifically for HPV 16. This review summarizes the existing evidence regarding the effect of HAART on HPV 16 infection, as this genotype may not be influenced by immunity level and explores its implications for Sub Saharan Africa. A comprehensive literature review was undertaken and quality assessment was carried out on the selected papers. Four cohort studies and three cross-sectional studies were identified for which the overall quality score assessment ranged from weak/moderate (Score of 1.8) to strong (Score of 3). The evidence yielded by our review was conflicting. Thus, the high heterogeneity between study populations and results did not allow us to draw any firm conclusions as to whether HAART has an impact on HPV 16 acquisition/prevalence. As only three studies were conducted in Africa, there are insufficient grounds for solid comparison between geographic regions. In light of inadequate data, HPV unvaccinated women on HAART should still receive more frequent follow-up.

## Introduction

In 2013, an estimated 35 million people were living with HIV worldwide [1]. Sub-Saharan Africa is home to only 12% of the global population, yet accounts for 71% of the global burden of HIV infection [2]. In 2007, the World Health Organization (WHO) included Invasive Cervical Cancer (ICC) to the “stage 4” HIV/AIDS classification of clinical staging and case definition of HIV for resource-constrained settings [3]. ICC is the most common female cancer in sub-Saharan Africa [4].

Human papillomavirus (HPV) is a sexually transmitted infection and high-risk (HR) HPV DNA has been shown to be present in 99.7% of cervical cancers worldwide [5]. Furthermore, HPV is considered the main causative agent in tonsil, tongue and squamous cell anal cancer as well as one of the major agents in squamous cell carcinoma of the vagina, vulva, penis, larynx, head and neck. Generally, HPV appears as the causative pathogen in 5% of all human cancers, with HPV 16 genotype as the most prominent contributor by far [6]. HR—HPV genotypes include HPV 16, 18, 31, 33, 35, 39, 45, 51, 52, 56, 58, 59 and 68.

Among the HR-HPV genotypes, HPV 16 and HPV 18 have the greatest oncogenic potential accounting for about 70% of all ICC [7]. HPV 16 tends to be persistent and, contrary to other genotypes, has been shown to be refractory to clearance in women on Highly Active Antiretroviral Therapy (HAART) [8].Furthermore, HPV 16 appears to have a higher replicative capacity, which is of epidemiological importance, as it may lead to an increased circulation and transmission rates [9].

Prophylactic HPV vaccines are likely to reduce the future burden of cervical cancer to a significant extent, particularly where screening is scarce, such as in Sub-Saharan Africa. The primary target group in most countries recommending HPV vaccination are adolescent girls aged 9–14. Prompted by the rapid effectiveness seen in many industrialized countries where the age range for HPV vaccination has been extended to cover women up to the age of 26, in 2016 the WHO revised its position and is hence recommending vaccination for this age group in resource-poor settings in order to step up HPV vaccine uptake. This, in turn, is expected to yield benefits at community level [10].

Furthermore, access to HAART in sub-Saharan Africa has greatly improved over the past decade, increasing life expectancy for women living with HIV [11]. In sub-Saharan Africa, the current standard recommendations for first-line adult antiretroviral therapy include two nucleoside reverse transcriptase inhibitors (NRTI) and one non-nucleoside reverse transcriptase inhibitor (NNRTI) [12]. Persons not responding to first-line regimens are usually switched to a cocktail of two NRTIs plus a boosted Protease inhibitor (PI) [13]. Following the latest WHO recommendation, more HIV infected persons may be initiating HAART regardless of the WHO clinical stage of HIV/AIDS and CD4 cell count [14]. Whilst in some European populations, a positive association between HIV infection and ICC has been documented in women on HAART [15], the picture is inconclusive in Africa [16].

A recent systematic review suggests that duration of HAART along with the CD4 count may reduce the prevalence of HR-HPV in sub-Saharan Africa [16]. A recent meta analysis reported that women on HAART had a lower prevalence of HR-HPV than those not on HAART (adjusted OR: 0·83, 95% CI: 0·70–0·99; I^2^ = 51%) [17]. This is likely to add an extra opportunity for primary prevention for unvaccinated young and older women. However, to be able to fine-tune a HAART-based prevention and determine an adequate HPV screening interval, the immuno-epidemiology of the most oncogenic HPV genotype still needs to be elucidated.

Whilst HPV genotypes other than HPV 16 are often better controlled by host’s immune responses, it is hypothesized that HPV 16 is better equipped to evade immune surveillance [18, 19]. Some studies have observed a higher relative prevalence of non-HPV 16 genotypes in severely immunocompromised women as well as in women with no access to HAART [19–21]. This may be attributed to a poor immune response against non-HPV 16 genotypes in severely immunosuppressed HIV infected women [18, 20]. It can be hypothesized that the variable effect of HAART in HIV infected women in Africa is contingent on the prevalence of HPV type 16 [22], which improved immunocompetence may not prevent. However, epidemiological evidence of this is still scattered and inconclusive. Should epidemiological evidence confirm that HAART has no impact on reducing HPV 16 prevalence, the current WHO recommendation of not rescreening women for at least 5 years, but within ten [23], among those screened negative will need to be revisited.

The primary objective of this review is to elucidate the impact and effects of HAART on prevalence/incidence of HPV 16 compared to other genotypes. The second objective is to highlight knowledge gaps for further research and specifically how they relate to the sub-Saharan African context.

## Methods

### Search strategy and selection criteria

We conducted this systematic review based on a pre-defined search protocol that conformed to the criteria set out by the PRISMA statement. PubMed, CINAHL, Global Health, Scopus, and EMBASE databases were searched between January 1997 and March 9, 2018 (S1 File).

### Search terms

The domains of the search terms were HPV 16 OR human papillomavirus genotype 16 AND acquisition OR incidence/detection OR prevalence AND Anti-HIV Agents OR Antiretroviral Therapy OR High Active OR antiretroviral OR ART OR HAART (S3 File)

### Study selection

We considered the following four components (Population, Intervention, Comparison, Outcome—PICO) to assess and categorize studies to be included in this review. The population involves HIV-positive women with HPV 16 infection (among other genotypes). The intervention examined in this study is the administration of HAART, defined as at least 3 antiretroviral drugs belonging to 2 different drug classes (Non-nucleoside reverse transcriptase inhibitors or nucleoside reverse transcriptase inhibitors or Protease Inhibitors) and, if possible, the duration of treatment. Women on HAART for at least 6 months are being compared to women not receiving HAART or just starting HAART. The outcomes of interest are: Prevalence or incidence or acquisition of HPV 16 for the two comparison groups.

We initially intended to conduct a systematic review and meta-analysis to answer the question on whether HIV positive women on HAART have higher probability to prevent HPV 16 infection than HIV-positive non-users of HAART. However, due to the scarcity and methodological heterogeneity of retrieved studies, and the lack of similarity of measure of effect of the various studies (Odds ratio vs Hazard ratio), pooling estimates would be inappropriate. We therefore re-oriented this study to a systematic review, without conducting a meta-analysis. The search strategy was designed by a medical librarian to identify studies reporting HPV 16 acquisition in women on HAART. We conducted this review on a pre-defined search protocol that conformed to the criteria set out by the PRISMA (S2 File) statement [24, 25]. (S2 and S3 Files)

### Data abstraction

All studies were independently reviewed and critically evaluated for inclusion by three authors (SM, ZN and RR). All data were extracted independently and in duplicate manner by the three investigators (SM, ZN and RR). Possible cases of disagreement were resolved on an individual basis via detailed discussion among the authors of the justification for exclusion/inclusion. The following items were recorded: first author, publication year, study design, type of sampling, sample collection method, study population, total sample size, HIV status, incidence/prevalence of HPV 16 and duration of HAART use, baseline of CD4 count in study population, sexual behavior, prevalence of multiple HPV genotypes, past history of STI and if the analysis included adjusting for confounding factors. The adjusted Odds ratio (a)OR, adjusted Hazard Ratio(a)HR and 95% Confidence interval (CI) were recorded and if the 95% CI did not include 1 and no p- value was given, it was considered significant.

### Data assessment

Because of the lack of randomized trials retrieved (due to ethical considerations), we considered prospective cohort/longitudinal studies to be of highest quality. We reported an overall score (from 1 to 3) based on our assessment of the overall quality of evidence for the following parameters:

1. Minimization of selection bias: (Score “3” = strong), the participants were likely to represent the target population; (Score “2” = moderate), somewhat likely; (Score “1” = weak), not likely to represent the target population.
2. Study design: (Score “3” = strong), a prospective study with a large sample size (> 200) [26]; (Score “2” = moderate), a prospective study with a sample size < 200 and a cross–sectional or case-control study with a sample size > 200; (Score “1” = weak), a cross sectional or case-control study with a sample size <200.
3. Confounders: (Score “3” = strong), controlled for all relevant confounders; (Score “2” = moderate), controlled for some confounders; (Score “1” = weak), control for confounders not specified.
4. Withdrawals, loss to follow-up and dropouts: (Score “3” = strong), description of a strategy to minimise the loss to follow-up, reported withdrawals and drop-outs (80–100% of participants completed the study) [27]; (Score “2” = moderate), reported withdrawals and drop-outs (60% of participants completed the study); (Score “1” = weak), withdrawal and drop-out rates not specified.

We assessed the methodological quality of evidence by calculating the mean of each study and attributing the mean one of the qualitative attributes, strong, moderate and weak.

### Ethical approval

No ethical approval was required as this was a systematic review (analysis of secondary aggregated data).

## Results

### Search results

On March 8, 2018, we retrieved 210 articles from PUBMED, CINAHL, Global Health, Scopus and EMBASE, of which 103 duplicate titles were identified. After screening the 107 remaining titles and abstracts, 11 were full text-screened and 7 were eventually included in this review. No disagreement between the three reviewers emerged on whether to include or exclude any given study.

### Study population

Studies varied in reporting, baseline of CD4 count in study population, sexual behavior, prevalence of multiple HPV genotypes, and past history of STI (Table 1).

**Table 1:** Study population characteristics.

First author (year)	Duration of HAART follow-up	CD4 count at HAART initiation	Lifetime partners	Regular Condom use (past 6 months)	Age (years.)	Multiple HPV genotypes	Past history of STI	currently sexually active
Blitz S (2013)	Median: 24 months for HIV + (IQR: 5–109 months)	Upon enrolment: Median; 336 cells/mm^3^ ((IQR, 180–515 cells/mm^3^)	HIV+ median: 5 (IQR: 3–12)	HIV+:187 (24.9%)	Median: 33 (IQR: 28–38)	71 (11.2%) had 2 genotypes 53 (8.4%) had 3 more genotypes		Not currently active HIV+: 264 (35.2%)
Konopnicki D (2013)	HPV 16: 45 months HPV18: 56 months Other HR: 57 months	In women with HPV 16: median CD4 count 439 CD4 cells/mm^3^ HPV 18 = 455 CD4 cells/ mm^3^	Not Reported	Not Reported	Median:42 (IQR: 35–48); HPV 16 median 34 and HPV 18, median: 42	11% had concomitant infection by other HR—HPV genotypes and HPV 16 or 18	Not Reported	Not Reported
Mane A (2012)	N/A	Mean (±SD) and median (IQR) CD4+ cell counts: 411 /μL ±214 and 372 /ul (241–556)	18.3% (51/278) had ≥2 sexual partners	Not Reported	Mean: 32.3 ± SD 5.3)	23/ 98, 23.4% had 2 HPV genotype and 4.1% had 3 genotypes	Women with HPV 16 had aOR [95%CI]: 1.12[0.47–2.66] of having a past STI	Not Reported
Shrestha S (2010)	Of the 227 HIV-positive participants, 100 were examined both before and after HAART initiation; 70 were examined only before HAART initiation; and 57 were examined only after HAART initiation, with overall median follow-up of 271 [IQR: 86.5–473] and 427.25 [IQR: 200–871] days respectively.	Median: 481 cells/mm^3^ [370–616]	Median: 6 sexual partners [IQR: 4–16]	Not Reported	Median: 17e [IQR: 16–18]	Median before HAAR initiation: 6 [IQR 3–10]; Median after HAART initiation: 7 [IQR: 4–11]	Not Reported	Not Reported
Van Aardt MC (2016)	Dichotomized ART > 12 months and ART 6–12 months	Patients not yet qualifying for HAART treatment (CD4 count >350 cells/μL); patients initiating HAART (CD4 count ≤350 cells/μL)	Not applicable	Not applicable	Range: 21–66	Mean: 2.56	Not Reported	Not Reported
Zeier DM (2015)	November 2009 to October 2011	Study Enrollment: mean 209 cells (SD:128) HAART initiation: 194 (SD:117 cells)	1–3 sexual partners: (64.7%); 4–6 sexual partners: (28.9%) 7 and more: 13 (6.4%)	Not applicable	Mean: 35.9 (SD: 9.26)	Not Reported	Not Reported	Not Reported
Firnhaber C. (2010)	N/A	CD4 count at HAART initiation was not reported. CD4 count was measured at study enrolment (median): <200CD4/mm^3^; 428 (42.4%) 200-500CD4/mm^3^: 464 (45.9%) >500CD4/mm^3^: 118 (11.7%)	Lifetime Sex partners>5: (42.8%) partners	Current condom use: The most commonly reported method of contraception Male condoms (75.4%),.	Median:34 (IQR: 18–65)	Not Reported	Not Reported	Not Reported

### Study design

A total of 3298 HIV infected women were included in the studies retrieved for this review. Sample sizes for these studies ranged from 225 to 1010 HIV infected women.

Seven studies were eventually included in this review. These studies varied by their design including prospective (n = 4) and retrospective cohort (n = 1) and cross-sectional (n = 2) studies. Four studies were conducted in developing countries (3 in sub-Saharan African countries and 1 in India) while the other three were in developed countries (USA, Canada and Belgium). Table 2

**Table 2:** Summary of findings.

First author (year)	Setting	Study design and sample size	Main exposure(s) and outcome(s) of interest	Main results concerning HAART and remarks	Confounding factors adjusted for
Blitz S (2013)	Canada	Prospective cohort study of 750 HIV-positive and 323 HIV-negative women	HIV, HAART use and SIL	HAART use did not significantly reduce the acquisition of HPV 16 or non HPV 16/18 oncogenic HR—HPV types (univariate HR: 0.52, 95% CI: 0.15–1.81) and (univariate HR: 0.94, 95% CI 0.41–2.16), respectively.	Not adjusted
Zeier DM (2015)	South Africa	Prospective cohort study of 300 HIV-positive women; 204 (68%) were initiated on ART during follow-up	Compare the effect of cART on each individual HPV genotype to the effect cART has on HPV-16	In the unadjusted model, the risk for detection of HPV was reduced by 77% (OR: 0.23, 95% CI: 0.15–0.37). cART significantly reduced the detection risk for cervical hrHPV (OR: 0.33, 95% CI: 0.24–0.44) and HPV-16 (OR: 0.50, 95% CI: 0.37–0.67). In the adjusted model, longer duration on cART had a stronger protective effect on HPV detection risk than CD4 T-cell count, also for HPV-16 detection risk (OR: 0.96, 95% CI:0.93–0.99; OR:0.94 (95% CI: 0.91–0.96) respectively.	Adjustment for months that elapsed since cART was first started), cervical HIV viral load, sexual behaviour and plasma HIV-RNA Level
Konopnicki D (2016)	Belgium	Prospective cohort study of 508 HIV-positive women	Prevalence of HR—HPV infection among HIV-positive women	In the univariate analysis, HPV 16 was significantly associated with age < 35 years or having a lowest median nadir CD4 cell count In multivariate analysis, being aged more than 35 years decreased the risk of being infected by HPV 16 (OR = 0.23; 95% CI: 0.06–0.89, p = 0.03 by logistic regression). At same median duration on ART, the prevalence of HPV 16 was not significantly different from that of other HR-HPV, 90% versus 86%, respectively.	Adjustment for age, CD4 count stage level and ART status
Mane A (2012)	India	Cross-sectional study of 278 HIV infected women (154 were on HAART)	Sociodemographic characteristics, bio-behavioural factors Outcome1: HPV infection Outcome2: CIN2+/CIN3+ prevalence	Lower CD4+ cell counts (AOR: 1.35, 95% CI: 1.09–1.67) and currently being on HAART treatment (AOR: 3.47, 95% CI: 1.40–8.59) were both independent factors significantly associated with presence of HPV 16	Adjusted for age, marital status, education, family income, parity, age at first sex, lifetime sex partners, past history of STI, tobacco use, CD4 count and HR-HPV
Shrestha S (2010)	United States	Longitudinal cohort study of 227 HIV-positive mostly African- American adolescents. 100 were examined both before and after HAART initiation	HAART use and HPV infection, clearance, and persistence in high-risk adolescents	Non-significant increase in HPV incidence after HAART initiation: before HAART 4.12/100 person-years (95%CI: 2.13–7.20) and 4.64/100 person-years (95%CI: 2.40–8.12) after HAART initiation	Internal control of potential confounders through case-cross over study
Van Aardt MC (2016)	South Africa	Cross-sectional study of 225 HIV-positive women	HAART and HR—HPV type infection	No significant effect was found for HPV 16. A 42.9% prevalence of HPV 16 among HAART non-users followed by 39.3%, 18.9% and 24.5% for women receiving HAART for < 6 months, between 6–12 months and >12 months, respectively	Adjusted for age and CD4 count in logistic regression analysis
Firnhaber C (2010)	South Africa	Cross-sectional study of 1010 HIV infected women	HIV-induced immune suppression and risk factors for HPV infection and cervical neoplasia among HIV-infected women	The prevalence of HPV type 16 was significantly more common among women with lower CD4 counts: 37.9% (33/87) vs. 5.9% (1/17); p = 0.01 among women with CD4 <200/mm^3^ and CD4>500/mm^3^ respectively.	No adjustment for confounding (only descriptive statistics)

### Quality assessment of studies

The overall quality of evidence pertaining to the association/impact of HAART on HPV 16 prevalence/incidence ranged from weak/moderate (Score of 1.8) to strong (Score of 3). One study had a score of 1.8 [28, 29] four studies had an average score between 2 and 3 [8, 29–31], hence the quality of their evidence was considered as “moderate to strong” and one study had a strong score of 3 [32].The weak ratings mainly resulted from lack of control of confounding factors (Table 3)

**Table 3:** Quality assessment of included studies.

First author (year)	Minimization of selection bias	Study design	Control of confounders	Loss to follow up	Overall methodological score and quality for research question
Blitz S (2013)	Strong	Strong	Weak	Strong	2.3 (moderate to strong)
Konopnicki D (2013)	Strong	Strong	Weak	N/A	2.3 (moderate to strong)
Mane A (2012)	Strong	Moderate	Strong	N/A	2.7 (moderate to strong)
Shrestha S (2014)	Strong	Strong	N/A	Weak	2.8 (moderate to strong)
Van Aardt MC (2016)	Strong	Strong	Weak	N/A	1.8 (weak to moderate)
Zeier DM (2015)	Strong	Strong	Strong	Strong	3.0 (strong)

### Systematic review results

#### HAART and HPV 16 prevalence in comparison with other HPV genotypes

All seven studies explored the impact of HAART on HPV 16 prevalence or incidence, of which four supported our hypothesis that HAART has no impact on reducing HPV 16 acquisition or prevalence. In a longitudinal cohort study of 750 HIV-infected women, Blitz (Canada, 2013) [8] found that HAART did not significantly reduce the acquisition of HPV 16 (univariate HR: 0.52, 95% CI: 0.15–1.81) or non-HPV 16/18 oncogenic HR—HPV types (univariate HR: 0.94, 95%CI: 0.41–2.16). In a multivariate analysis, (Belgium, 2013) [29] at same median duration on ART, the prevalence of HPV 16 was not significantly different from the prevalence of other HR- HPV, 90% versus 86%, respectively.

In a prospective cohort study on adolescents, (USA, 2010) [30] HAART did not have an effect on vaccine-type HPV incidence, clearance, and persistence. Whilst HPV 16 was the most prevalent before HAART initiation (17%, 95% CI: 11–22%), HPV 53/66 after HAART initiation period had a higher prevalence (18%, 95% CI: 12–24%). Within a case-crossover sub-study of the same prospective cohort study, it is noteworthy that there was an insignificant increase after HAART initiation: 4.12/100 person-years (95% CI: 2.13–7.20) before HAART initiation and 4.64/100 person-years (95% CI: 2.40–8.12) after HAART initiation. Van Aardt et al (South Africa, 2016) [28] in a cross-sectional study, reported a 42.9% prevalence of HPV 16 among HAART non-users followed by 39.3%, 18.9% and 24.5% for women receiving HAART treatment for < 6 months, between 6–12 months and >12 months, respectively. They showed that in terms of effectiveness, HAART treatments exceeding 12 months in duration was were negatively associated with HPV types 33, 59 and 82, and positively associated with HPV 73. No significant effect was found for HPV 16.

We identified three studies, which refute our hypothesis. Zeier (South Africa, 2015) [32], in the univariate analysis, reported that HAART in a univariate model for detection of any HPV genotype, and HAART status (receiving HAART or not) as the only predictor, HAART reduced the risk of HPV presence by 77% (OR: 0.23, 95% CI: 0.15–0.37). HAART significantly reduced the detection risk for cervical HR- HPV presence (OR: 0.33, 95% CI: 0.24–0.44) and HPV-16 (OR: 0.50, 95% CI: 0.37–0.67). In the adjusted model, longer duration on HAART had a stronger protective effect on HPV detection risk than CD4 T-cell count (OR = 0.96, 95%CI: 0.93–0.99), and this also applied to HPV-16 presence detection risk (OR = 0.94; 95%CI 0.91–0.96). Firnhaber et al (South Africa, 2010) [31] reported that the prevalence of HPV type 16 was more common among women with lower CD4 counts 37.9% (33/87) for CD4 200/mm3 vs. 5.9% (1/17) for CD4 500/mm3 (P = 0.01). These results suggest that, within the African continent, HPV 16 may not be better at evading host immune responses than other HPV types, as previously suggested, but is simply more able to thrive in an immunocompromised host [19]. HPV 16 prevalence also drops, as other HPV types do, when the immunity is re-established (eventually by HAART). Mane (India, 2012) [33] reported that a lower CD4+ cell counts (AOR: 1.35, 95%CI: 1.09–1.67) and an ongoing HAART treatment (AOR: 3.47, 95%CI: 1.40–8.59) were both statistically significant factors associated with presence of HPV 16. They were however not associated with presence of non-HPV 16 carcinogenic types. (OR = 0.94; 95%CI 0.91–0.96).

## Discussion

The findings from this review are conflicting and contradictory. Therefore, a conclusive answer on the impact and effect of HAART on the incidence and prevalence of HPV 16 is not possible. The overall quality of evidence pertaining to the association/impact of HAART on HPV 16 prevalence/incidence ranged from weak/moderate to strong, which further reduces our capacity to explore the association.

Our study builds on the recent global meta-analysis [17], which explored the pooled effect of HAART on aggregate HR—HPV prevalence, by identifying other studies which exclusively focused on comparing incidence or prevalence of the most virulent, carcinogenic and aggressive HPV genotype (HPV 16) to other HR—HPV genotypes.

Moreover, given the fact that only three studies took place in Sub Saharan Africa, we are limited in our capability to compare findings between this geographical region and others. This was exacerbated by the high degree of heterogeneity in quality, reporting, setting, study population and results. The baseline CD4 count was mostly given upon enrollment of in the study, but not when HAART was initiated. And in only one study [30], the median CD4 count at baseline was known in HPV infected women on HAART with HPV 16.

Furthermore, only one study examined past history of STIs [33]. A meta-analysis suggested a significant association between bacterial vaginosis (BV) and HR—HPV genotypes [34]. HPV 16 and its phylogenetically-related genotypes have also been linked with BV. Studies have suggested a significant association between BV and HPV 16 in female sex workers in Spain [35], and a marginal association between BV and HPV 58, a HPV 16- related type in Kenya [36].

Moreover, only one study [8] examined regular condom use in the past six months and recent sexual partners, which may be more relevant confounders to adjust for than the number of past sexual partners, as previous HPV 16 infection may have cleared up in the interim. Due to variations in intervention duration, sample size, study location, individual variations in CD4 counts and studied the populations studied, evidence on the impact of HAART and HPV acquisition in HIV-infected women remains inconclusive. No study examined HIV viral load during HAART treatment.

### Strengths and limitations of this study

The major strength of this study is that there has been a thorough attempt to search five databases and retrieve all the relevant existing literature. Nonetheless, this study does have limitations as well, including our incapacity to carry out a meta-analysis due to the heterogeneity of study designs and their measurement of effect.

### Public health and clinical implication

In light of the inconclusive findings of the review, unvaccinated HIV-infected women may still require more regular monitoring than once every three years if tested negative by VIA or cytology, as recommended by the WHO for this population [37], despite recent findings that HAART may reduce HR—HPV prevalence [17]. In turn, these findings underscore the importance of primary prevention of HPV-16 acquisition through prophylactic vaccines due to the scarcity of evidence on whether HAART is specifically able to confer a protective effect against HPV 16 as it does against other HR—HPV genotypes [17].

### Research gaps

A major gap is to elucidate the HPV 16 Basic Reproductive Number (R0) in various types of populations, which may reveal what percentage of the population needs to be vaccinated. In light of the rollout of HAART at a higher CD4 count in sub-Saharan Africa, it is imperative that the prevalence of HPV 16 in HIV-infected women per sub-Saharan country be better established. Knowledge about the specific HPV type distribution in the HAART era is crucial to guide HPV-based screening intervals and the feasibility of catch-up campaigns focusing on older HIV-infected girls.

Recent literature suggests that the lesser impact expected of the HPV vaccine in older HIV-infected girls, due to a potential higher risk of prior exposure to vaccine-targeted genotypes, may be compensated by the potential of the HPV vaccine to prevent recurrence of CIN 2–3 among women treated for HSIL [38]. In this population, the incidence of disease recurrence after treatment varies widely from 25 to 55% at 12 months [39]. In addition, research is required to explore whether HAART initiation at a less immunocompromised state can prevent subsequent HPV 16 infections by generating antibodies and hence providing protection against new infections.

In light of the increasing number of HIV-infected women accessing HAART at a less immunocompromised state, the impact of different levels of immune reconstitution conferred by HAART on HPV 16 acquisition as a stand-alone or as co-infection with other HR—HPV genotypes should be elucidated. Hence, it is imperative that more studies on the sub-Saharan continent be undertaken and disaggregated baseline data be collected by HPV 16 status in order to adjust for potential confounders.

As the prevalence of BV in sub-Saharan women is among highest in the world [40], it is pivotal that the association between BV and HPV 16 in women on HAART is elucidated and whether it would be cost-effective to launch catch-up campaigns among HIV-infected women below 26 years of age.

Rollout of HAART in sub Saharan Africa has brought forth concomitant increases in drug resistance rates, which can be attributed to the empirical first and second line antiretroviral regimens and the clinical or immunological definitions of treatment failure without plasma viral load monitoring [12]. Data on second-line failure and development of PI resistance in sub-Saharan Africa are scarce [41]. As third line drug options, such as integrase inhibitors, including dolutegravir, are expected to increase [42], longitudinal studies are required to elucidate the impact of HAART on HR—HPV genotypes as well as on HPV 16 acquisition in HIV-infected women.

## Conclusion

Our limited data does not enable us to yield conclusive findings. To err on the side of caution, our data suggests that unvaccinated women on HAART may require more frequent follow-up. The scarcity of data on acquisition of specific HPV genotypes by HAART regimens is reflective of the lack of data found on the immuno-epidemiology of oncogenic genotypes and their synergistic interactions prior and after initiation of HAART. Apart from recent literature suggesting a protective effect of HAART against HR—HPV infection, further research on the impact of HAART on HPV 16 infection is needed with a view to designing an optimal screening and vaccine strategy in HIV-infected women in Sub Saharan Africa.

## Supporting information

S1 File Search strategy. (TIFF)

S2 File PRISMA checklist. (TIFF)

S3 File Flow diagram. (TIFF)

## Abbreviations

HAART: Highly Active Antiretroviral Therapy
HIV: human immunodeficiency virus
HPV: Human papillomavirus
ICC: Invasive cervical cancer
WHO: World Health organization
BV: Bacterial vaginosis
OR: Odds ratio
(a)OR: adjusted odd ratio
HR: Hazard ratio
(a)HR: adjusted Hazard ratio
IQR: Interquartile range
