# Supplementary figures and images for: Relationship between Highly Active Antiretroviral Therapy (HAART) and human papillomavirus type 16 (HPV 16) infection among women in Sub-Saharan Africa and public health implications: A systematic review
Source: PLoS One. 2019 Mar 11;14(3):e0213086. doi: 10.1371/journal.pone.0213086 (PMC6411162; doi:10.1371/journal.pone.0213086)

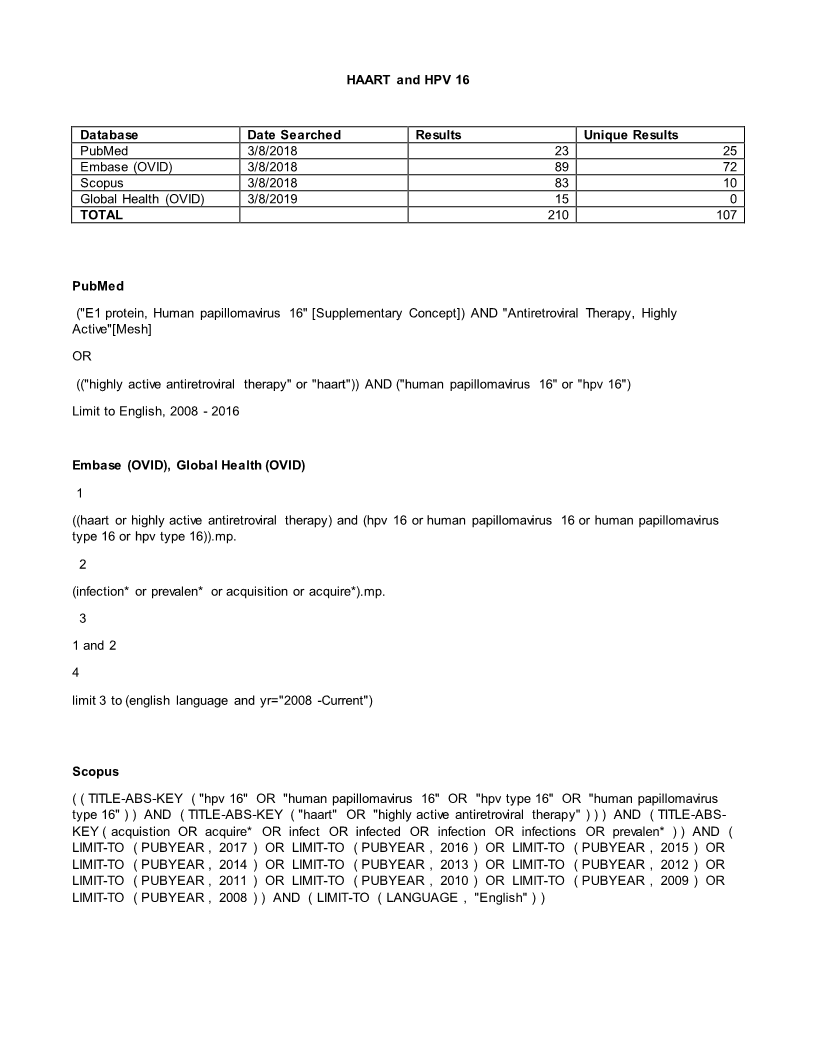

Supplement: S1 File — (TIFF) [file pone.0213086.s001.tiff]

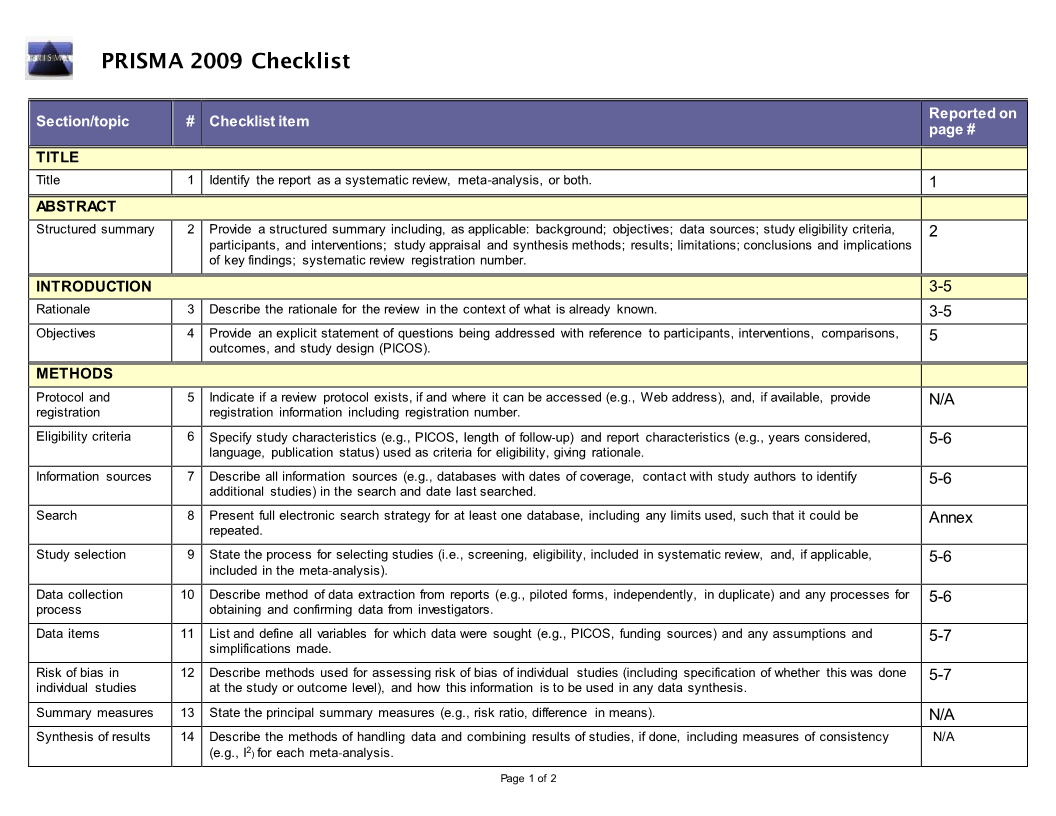

Supplement: S2 File — (TIFF) [file pone.0213086.s002.tiff]

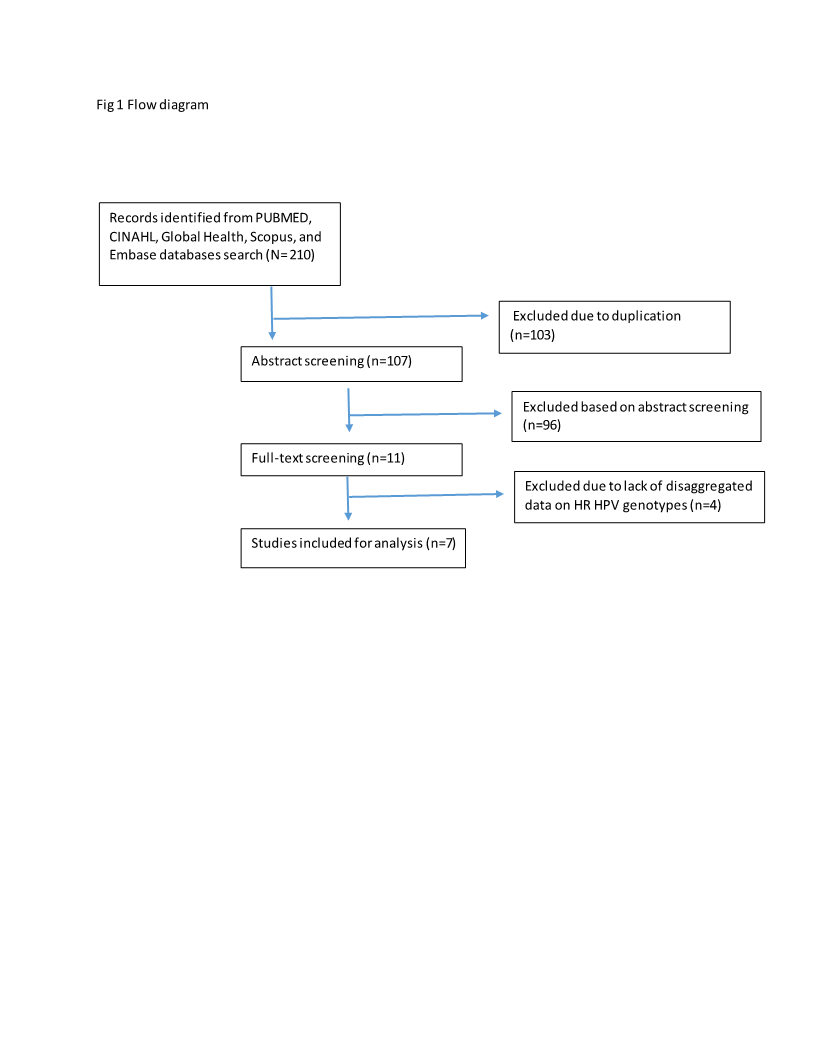

Supplement: S3 File — (TIFF) [file pone.0213086.s003.tiff]
